# Autophagy in neurodegeneration and development

**DOI:** 10.1016/j.bbadis.2008.06.010

**Published:** 2008-12

**Authors:** Ashley R. Winslow, David C. Rubinsztein

**Affiliations:** Department of Medical Genetics, Cambridge Institute for Medical Research, Wellcome Trust/MRC Building, Addenbrooke's Hospital, Hills Road, Cambridge CB2 0XY, UK

**Keywords:** Autophagy, Neurodegeneration, Development, Endosomal sorting complexes required for transport (ESCRT), Lysosomal storage disorder (LSD), Dynein

## Abstract

Efficient protein turnover is essential for the maintenance of cellular health. Here we review how autophagy has fundamental functions in cellular homeostasis and possible uses as a therapeutic strategy for neurodegenerative diseases associated with intracytosolic aggregate formation, like Huntington's disease (HD). Drugs like rapamycin, that induce autophagy, increase the clearance of mutant huntingtin fragments and ameliorate the pathology in cell and animal models of HD and related conditions. In *Drosophila*, the beneficial effects of rapamycin in diseases related to HD are autophagy-dependent. We will also discuss the importance of autophagy in early stages of development and its possible contribution as a secondary disease mechanism in forms of fronto-temporal dementias, motor neuron disease, and lysosomal storage disorders.

## 1 Protein degradation systems

The ubiquitin-proteasome system (UPS) and the autophagy-lysosome pathway are the two main degradative pathways in eukaryotes. Both of these systems are responsible for the efficient degradation and turnover of proteins within the cell. Failure of either the UPS or autophagy has been associated with disease while the upregulation of these processes has been shown to ameliorate certain disease pathologies.

The proteasome is responsible for the selective degradation or recycling of short-lived cytosolic and nuclear proteins, but also regulates the turnover of some long-lived proteins. Attachment of a chain of at least four covalently bonded ubiquitin molecules to a protein targets it for degradation by the proteasome. The proteasome, a 2000 kDa, multisubunit protein complex has a hollow barrel that contains its proteolytic components. Because of the small size of the proteasome's proteolytic core, client proteins generally require unfolding prior to degradation. Due to the size of the narrow barrel of the proteasome and the specificity of the process, many proteins are unable to be degraded by the UPS, including oligomers or proteins in organelles [1].

Macroautophagy, more commonly referred to as autophagy, is responsible for the bulk degradation of long-lived cytosolic proteins and organelles. Unlike proteasomal degradation, autophagic degradation is thought to be largely non-specific. Degradation of macromolecules or organelles by autophagy requires the formation of double-membrane vesicles called autophagosomes and subsequent fusion of autophagosomes with lysosomes. This fusion produces an autophagolysosome within which the cargo is degraded by acidic lysosomal hydrolases.

## 2 Early studies in yeast

Despite an advanced understanding of the UPS, many aspects of autophagy remain largely unknown. The discovery of pathways that induce autophagy has contributed to the progression of our understanding of autophagy as a degradative pathway and its implications in human health.

Autophagy is induced under starvation conditions, while it is inhibited under nutrient-rich conditions [2,3]. An increase in protein degradation during starvation allows the cell to survive stressful conditions by providing nutrient turnover when extracellular nutrients are scarce. Many of the earliest studies into the genetic control of autophagy used viability during starvation as an assay to screen for autophagy mutants in yeast [2,4–7]. Additionally, these screens and a number of other labs exploited the simple yeast system to identify new autophagy genes through a combination of morphological and biochemical tools. Morphologically, the light microscope was used to visualise the accumulation of vacuolar structures within cells under starvation conditions, while biochemical analysis of the accumulation of fatty acid synthase, an autophagy substrate, allowed researchers to quantify autophagy function.

These early screens provided a foundation for the understanding of autophagy in yeast and higher eukaryotes by identifying key proteins and interactions. From the assessment of protein interactions and autophagosome morphology, autophagy degradation has been found to consist of six main steps: induction, cargo selection and packaging, autophagosome formation and completion, recycling of autophagy regulators, fusion of the autophagosome with the lysosome, and cargo degradation. These steps rely on the function of key autophagy genes (Atg genes) [8] and two highly conserved ubiquitin-like conjugation reactions, Atg12–Atg5 [9,10] and Atg8-phosphatidylethanolamine (PE) conjugation [8,11,12]. The Atg12–Atg5 complex can additionally bind Atg16 which mediates the formation of tetrameric and octameric complexes of Atg12–Atg5–Atg16 [13,14]. The function of this large molecular structure is important to autophagosome membrane formation and has been implicated in stabilisation of the Atg8–PE complex, the other important conjugation system [15]. Atg8, referred to as microtubule associated protein 1 light chain 3 (LC3) in mammals, undergoes a similar but reversible conjugation reaction with phosphotidlyethanolamine (PE). This reaction lipidates the cytosolic LC3-I isoform, forming LC3-II, an autophagosome membrane-bound protein [16]. LC3-II is the only protein known to remain attached to the membrane after complete formation of the autophagosome and therefore is a robust marker of autophagosome number. Changes in steady-state LC3-II levels provide both a surrogate for autophagosome number and an idea as to where these effects occur in the progression of autophagy.

## 3 Autophagy in development

In light of the importance of the degradative pathways in mammalian disease, a branch of autophagy research has studied the role of autophagy in normal development and maintenance of health. It has been shown that autophagy is important during critical developmental stages in which nutrients are restricted. Termination of the direct fetal nutrient supply from the mother presents a stressful situation for the newborn infant. During this transition period, autophagy provides the necessary nutrients to the infant through increased turnover of proteins. This has been shown experimentally in a transgenic mouse model expressing GFP fused LC3 to visualise autophagosomes in vivo [17]. In these mice, autophagosome formation was increased naturally within 30 min after birth, peaked around 6 h after birth, and declined back to basal levels within 24 to 48 h. These observations support the role of autophagy as a means of increasing newly-available nutrients in a nutrient deprived environment. Thereby, neonates are able to cope with severe starvation after birth by the induction of autophagy and turnover of proteins.

To assess the specific role of autophagy, Mizushima's and Tanaka's groups studied the effect of starvation during this critical period in Atg5 and Atg7 knockout mice, i.e. autophagy-deficient mice. These mice develop normally with only a slightly lower birth weight for the Atg5^−/−^ mice and a significant weight reduction in the Atg7^−/−^ mice. Atg5 [17] and Atg7 [18] knockout mice die within one day of birth. Under forced starvation conditions, Atg5 and Atg7 knockout mice die within half the time (12–13 h) of their Atg5^+/−^ or wildtype littermates (21–24 h). These experiments support the role of autophagy in normal developmental processes that occur in the absence or diminished levels of nutrient supply, lending further importance to the need for the functional turnover of amino acids.

As a systemic deficiency of autophagy genes in these mice causes neonatal death within a day of birth, conditional autophagy-deficient mice were generated to assess the role of autophagy in homeostasis of various tissues later in development. In the absence of a predisposition to genetic disease, both Atg5 and Atg7 conditional knockout mice and ubiquitous knockout mice develop ubiquitinated protein aggregates and varying degrees of tissue deterioration. Conditional Atg5 and Atg7 knockout mice suffer not only from nutrient deprivation during starvation periods but also develop dysfunctional hepatic tissue and progressive neurodegeneration [17–19]. Loss of Atg5 specifically in neural tissue of 10.5 day old mice results in growth retardation, weight loss, an accumulation of ubiquitin-positive proteins, motor impairment and progressive neurodegeneration. Within hepatocytes, conditional Atg5 knockout mice develop diffuse abnormal protein deposits which progress to inclusion bodies 16 days after induced knockdown [20]. Similar conditions developed in hepatocytes of Atg7 conditional knockout mice. A nearly six-fold enlargement of the liver was accompanied by the accumulation of ubiquitin-positive inclusions within the cytosol of hepatocytes and defective cellular organelles such as mitochondria, ER and peroxisomes.

A recent study analysed the role of p62/A170/SQSTM1 in relation to inclusion formation in autophagy-deficient mice. Loss of p62 in autophagy-deficient mice decreased levels of polyubiquitinated proteins and inclusions. The authors suggested that this was because p62 was a key mediator of inclusion formation [21]. Interestingly, similar conclusions were deduced from a parallel study of the *Drosophila* p62 orthologue in autophagy-deficient flies [22].

Additional characterisation of autophagy deficiency has been performed in Atg7 *Drosophila melanogaster* mutants. Although Atg7 mutant flies are morphologically normal, they are sensitive to stressful conditions and show a decreased level of cell death-induced autophagy during metamorphosis, a necessity during normal development [23]. Increased lethality and a decrease in GFP-LC3 levels in Atg7 deficient flies indicated that autophagy deficiency was deleterious during stress-inducing conditions such as starvation, oxidative stress and metamorphosis. Similar to Atg7-deficient mouse models, these flies develop large inclusion bodies and accumulate ubiquitinated proteins within neurons, in addition to a shortened life span under unstressed conditions. Motor function, as measured in climbing assays, progressively worsens in these flies, an indicator of reduced neuronal function.

Studies in *Drosophila* have also contributed to an understanding of the roles autophagy may play in ageing. Atg8a mutant flies have reduced lifespan, while transgenic overexpression of Atg8a extends the fly lifespan by 56% and increases its resistance to oxidative stress and reduces the accumulation of ubiquitinated proteins [24].

## 4 Autophagy deficiency: a secondary disease mechanism in neurodegeneration

Autophagosomes need to ultimately fuse with lysosomes. The endocytic pathway is necessary for maturation of autophagosomes via the fusion of autophagosomes with endosomes, prior to autophagosome–lysosome fusion [25]. The cytoskeleton maintains the spatial organisation of autophagy by conducting the trafficking of organelles involved in different interactions during autophagy. Many of the pathways that contribute to autophagy have only recently been identified and it is expected that autophagy interacts with numerous other systems either through its own progression or by the intracellular signals that control autophagic activity. From this understanding of autophagy as a dynamic integrator of numerous signalling pathways, it is evident that disruptions in the functionality of other pathways are likely to disrupt autophagy and therefore cellular homeostasis. The most recent research within the field has raised the idea of the role of compromised autophagy in the progression of disease. A number of diseases have been shown to manifest a deficiency in autophagy, identifying autophagy as an important secondary disease mechanism.

## 5 Dynein

The relay of signals within and between cells is dependent upon efficient internal transport. Neuronal cells are particularly dependent on the accurate and timely conversion of external stimuli to an intercellular response via the movement of internal cargo. Directed movements over long distances, such as that seen in motor neurons, are dependent upon microtubules via dynein and kinesin motors. Dyneins move cargo centripetally towards the minus-end of microtubules, i.e. towards the microtubule organising centre near the nucleus. Plus-end-directed kinesins move cargo in the opposite direction, centrifugally, outwards into the cytoplasm and the plus-end of microtubules (reviewed in [26]).

Autophagic flux is microtubule-dependent, as depolymerisation of microtubules with nocodazole inhibits the fusion of autophagosomes with lysosomes [27,28]. Depolymerisation of microtubules results in a decrease in the clearance of autophagy substrates [29]. Under these conditions, autophagosome maturation [30,31] and autophagosome–lysosomal fusion are decreased, as autophagosomes are unable to shuttle from the cell periphery to the MTOC via microtubules [32]. Knock down of dynein shows similar effects, suggesting that dyneins are the key motor proteins that traffic autophagosomes along microtubules towards lysosomes [32]. It is unclear how dyneins are connected to the autophagy machinery. However, HDAC6, a cytosolic histone deacetylase-like protein is associated with microtubules and appears to be a modulator of autophagy [33,34]. While its mode of action remains unclear, it is possible that it is involved in autophagosome trafficking on microtubules.

A number of mutations affecting microtubule transport have been implicated in the development of motor neuron diseases (MND) in mouse models [35–37] and humans [38]. Motor neuron disease refers to a group of sporadic and familial diseases characterised by the degeneration of motor neurons. Disruption of retrograde axonal transport of cargo by overexpression or depletion of dynein complex components in transgenic mice results in the progressive degeneration of motor neurons and the formation of inclusions [35,37]. The pathology and symptomatic progression of the disease in these transgenic models mimic those seen in some MND patients. Interestingly, the presence of aggregates in MND models may hint at a deficiency in autophagy as a secondary disease mechanism in these disorders. As disruption in dynein-dependent transport is implicated in the cause of this group of diseases, it is likely that the aggregation of substrates seen in mouse models could, at least, in part result from the disruption of the microtubule-dependent movement of autophagosomes, autophagosome maturation, and autophagosome–lysosome fusion. Indeed, an increase in autophagosome number and LC3-II levels can be observed in dynein-deficient transgenic mice [29,39]. Further research will be needed to clarify the significance of autophagy dysfunction in MND, particularly in forms not due to primary mutations of the dynein machinery.

## 6 The ESCRT complex

The turnover and recycling of integral membrane proteins from the plasma membrane is carried out largely by different functions of the endocytic pathway. Simple sorting by endosomes recycles proteins, returning them to the plasma membrane. A more intricate degradative system utilises the multivesicular body (MVB), a specialised vesicular structure generated by invagination of the endosomal membrane to form lumenal vesicles. The fusion of MVBs with lysosomes releases the vesicles into the acidic lumen of the lysosomes within which hydrolases degrade the vesicles and their cargo. The sorting of integral membrane proteins into the MVB pathway is dependent upon monoubiquitination of the protein, a signal that destines the protein for incorporation into the lumenal vesicles of MVBs [40–44]. The fidelity of this process from protein sorting to endosomal-lysosomal fusion is maintained by the sequential interaction of four complexes termed the endosomal sorting complexes required for transport (ESCRT complexes). The specific interactions of these complexes are necessary for the formation of the MVB and proper progression of endosomal–lysosomal fusion (reviewed in [45]). Each of the ESCRTs is recruited to their particular function through multiple interactions with proteins, membranes, endosomes, and other ESCRTs. Early cargo sorting is carried out by the interactions of ESCRT-0, -I, and -II with ubiquitinated cargo. ESCRT-III is required for disassembly of the ESCRT complex and recruitment of de-ubiquitilating enzymes (DUBs) to remove ubiquitin from the cargo prior to degradation. The final actions of ESCRT-III concentrate cargo in late endosomes.

In addition to known roles in HIV budding [46] and tumour suppression [47–49], the ESCRT machinery has been implicated recently in neurodegenerative disorders, such as frontotemporal dementia linked to chromosome 3 (FTD3) [50] and amyotrophic lateral sclerosis (ALS) [51,52]. Despite the association of specific ESCRT machinery mutations with neurodegenerative disorders, the mechanism of disease remains largely unknown. A point mutation in the ESCRT-III subunit CHMP2B that alters a splice-site within the gene was found to be associated with a rare familial autosomal dominant form of FTD3 [50]. Additionally, two different point mutations in CHMP2B have also been associated with a non-SOD1 form of ALS [52,53].

Expression of a deletion mutant of CHMP2B, synonymous to the splice mutant, increases neuronal and dendritic loss through an apoptosis-independent pathway [54]. As autophagosomes are thought to fuse with endosomes or MVBs prior to fusion with lysosomes, recent studies have looked at the effect of the CHMP2B deletion mutant on autophagy [54,55]. Within cell and fly models, expression of this deletion mutant increased LC3-II levels, caused an accumulation of autophagosomes, and decreased the formation of MVBs. Experimental characterisation of CHMP2B and a mutant form of SKD1 [56] (first shown with the yeast homologue Vps4 [57]) that prevents dissociation of the ESCRT-III complex prior to the next round of endocytic cargo sorting, indicates that the proper dissociation of the ESCRT-III complex is critical to both autophagosome maturation and proper fusion of autophagosomes with lysosomes. ESCRT dysfunction has been shown to cause an accumulation of autophagosomes in cortical neurons and flies [54].

The effect of ESCRT dysfunction on autophagy has been studied by the expression of mutants or the knockdown of genes in all three complexes and associated proteins: (associated proteins (SKD1/Vps4 [56,57], fab1 [58]), ESCRT-III-CHMP2B [54,55], mSnf7-2/Vps32 [54,58], Vps24 [55]), (ESCRT-II-Vps25 [58], Vps22 [55]), and (ESCRT-I-Vps28 [58], Tsg101/Vps23 [55]). Data accumulated from studies of loss-of-function of various ESCRT genes in a wide range of cell types, suggest that these defects result in deficient maturation of autophagosomes or in their inability to fuse with lysosomes and endosomes. Autophagosomes accumulate without degradation of their cargo leading to neurodegeneration in many cases. Additionally, many of these tissues display an increase in polyubiquitinated inclusions. It remains unclear whether this deficiency in autophagy is a direct effect of the disruption of the ESCRT complex on autophagosomes or indirect via endosomes and lysosomes. Overall, recent research suggests that the deficiency in autophagy caused by ESCRT dysfunction contributes to the neurodegeneration in FTD3 and ALS as a secondary disease mechanism.

## 7 Lysosomal storage disorders

The role of lysosomes in autophagy has been well characterised from an early point in autophagy research [59]. Fusion with lysosomes is necessary for the degradation of autophagy cargo by the lysosomal acidic hydrolases. Without this fusion event autophagosomes accumulate with undegraded cargo, thus increasing the toxic species within cells. Recent research suggests that this defect may be characteristic of a group of neurodegenerative diseases referred to as lysosomal storage disorders [60].

Lysosomal storage disorders (LSDs) are a group of at least 40 neurodegenerative or myopathic diseases defined by the accumulation of undegraded lysosomal substrates within the lysosomal lumen [61]. Generally speaking, LSDs are monogenic diseases caused by mutations that lead to complete or partial dysfunction of lysosomal proteins (typically lysosomal hydrolases). It has been estimated that lysosomes contain 50–60 different hydrolases [62] that are active within the acidic pH of the lysosomal lumen. In addition to a number of hydrolases, multiple integral membrane proteins have been identified, most with no known function [63]. Despite the large differences in the pathology of these diseases, at a molecular level all of these diseases result in the accumulation of undegraded cargo both within the lysosomal lumen and outside lysosomes. As it is clear that this accumulation affects numerous other pathways, it is not known which species and pathways contribute most significantly to the progression of these diseases (reviewed in [61,64]).

The current understanding of autophagy and its dependence upon lysosomes has led researchers to study the effects of LSDs on both basal autophagy and the induction of autophagy. Fusion of autophagosomes with lysosomes is necessary for the degradation of cargo by the lysosomal acidic hydrolases. Without this fusion event autophagosomes and their cargo accumulate, thus increasing toxic species within cells. A recent study [60] identified an inhibition of autophagy-dependent protein degradation in a mouse model of multiple sulfatase deficiency (MSD) and mucopolysaccharidosis type IIIA (MPS-IIIA). MSD, an aggressive neurodegenerative disorder that results in death, is caused by a mutation in the sulfatase modifying factor 1 (SUMF1) gene which encodes the formylglycine-generating enzyme (FGE) [65,66]. FGE is required for posttranslational activation of sulfatases. Without this modification, sulfatase activity is impaired and the enzymes are therefore unable to degrade lysosomal contents. In addition to an accumulation of lysosomes and undegraded lysosomal substrates, MSD mice accumulated autophagosomes. Elevated LC3-II levels detected by immunoblotting and LC3-positive vesicles in neuronal tissue confirmed an accumulation of autophagosomes. Analysis of cell lines derived from MSD mice and their wildtype littermates showed a decrease in the colocalisation of the lysosomal marker, LAMP1, and LC3 in MSD mouse embryonic fibroblasts (MEFs). This indicates that the accumulation of autophagosomes results from failed fusion of autophagosomes with lysosomes. The mutation of SUMF1 in MSD mice prevents both the degradation of lysosomal cargo and the fusion of autophagosomes with lysosomes. The accumulation of the autophagy substrates within MSD MEFs further supports a deficiency in autophagy as a secondary disease mechanism in LSDs. Brain tissue of MSD mice showed an accumulation of ubiquitinated aggregates.

These examples suggest that autophagy deficiency may be an important secondary disease mechanism in a range of conditions.

## 8 Induction of autophagy as a therapeutic for neurodegenerative disorders

Intracellular protein misfolding/aggregation are features of many late-onset neurodegenerative diseases, called proteinopathies. These include Alzheimer's disease, Parkinson's disease (PD), tauopathies, polyglutamine expansion diseases – such as Huntington's disease (HD) – and various spinocerebellar ataxias (SCAs), like SCA3. Currently, there are no effective strategies to slow or prevent the neurodegeneration or muscular dystrophy resulting from these diseases in humans. Due to the size of the narrow barrel of the proteasome and specificity of the process, most large aggregate-prone proteins are precluded from entering the structure [1]. We discovered that aggregate-prone species of proteins like mutant huntingtin have a high dependency on autophagy for their clearance, while wild-type species do not rely on autophagy for their degradation [67–71]. Inhibition of autophagy by chemical regulators, such as bafilomycin A1 and 3-methyladenine, decreases the clearance of aggregate-prone proteins such as mutant huntingtin (that causes Huntington's disease) or mutant forms of alpha-synuclein that cause familial Parkinson's disease. This results in an increase in the number of huntingtin aggregates in mammalian cells [67,68,71].

The discovery of target of rapamycin (TOR) as a pivotal regulator of autophagy induction in eukaryotes [72], pushed the scope of autophagy research from its fundamental function in cellular health and homeostasis to a possible therapeutic. An understanding of the function of TOR function as a negative regulator of autophagy allowed researchers to exploit rapamycin, an inhibitor of TOR, as a means to induce autophagy. Rapamycin induces autophagy in species from yeast to mammals and appears to have this effect in all mammalian cells tested to date. Chemical induction of autophagy by rapamycin enhanced clearance of aggregate-prone proteins and reduced the number of aggregates [71]. Additionally, amelioration of neurodegenerative symptoms by autophagy induction has been seen in both a *Drosophila* HD model and transgenic HD mice [70]. It is likely that this autophagic clearance is specific to the smaller oligomeric and monomeric precursors of aggregates, not the large inclusions [73].

These findings are replicable in other proteinopathy models. For instance, rapamycin reduces toxicity and enhances clearance of aggregate-prone proteins, like a polyalanine expansion protein and tau (associated with front-temporal dementia or tauopathy). These results provide proof-of-principle in the ability of autophagy to clear aggregate-prone proteins [52,74] and suggest a druggable target pathway which may ameliorate symptoms or prevent disease. Importantly, the effects of rapamycin in fly models of these diseases appear to be autophagy-dependent, as rapamycin had no effects on proteinopathy toxicity in flies expressing these mutant proteins on a background of reduced activity of different autophagy genes [52,75].

Unfortunately, long-term use of rapamycin can cause adverse side effects such as poor wound-healing and general immunosupression. Mammalian target of rapamycin (mTOR) regulates a range of pathways (such as translation of certain proteins, cell division, etc.), and its major side-effects can be attributed to autophagy independent pathways. Also, how mTOR regulates mammalian autophagy is still not understood. Further insight into how this occurs will hopefully provide safer, more specific druggable targets suitable for long-term treatment.

Currently, there exist very few pharmacological treatment options for neurodegenerative proteinopathies. The disaccharide trehalose has also been shown to reduce aggregation and induce autophagy in an mTOR-independent manner, but this process and the pathway through which it acts remain unknown [76,77]. Recently, an mTOR-independent pathway for autophagy induction has been discovered: inhibition of inositol monophosphatase (IMPase) reduces free inositol and myoinositiol-1,4,5-triphosphate (IP3) levels which lead to an upregulation of autophagy. Lithium, carbemazepine and valproate, drugs used to treat a range of neurological and psychiatric conditions, induce autophagy via this pathway. Like rapamycin, these drugs increase the clearance of aggregate-prone proteins like mutant huntingtin and have beneficial effects in fly models of HD [78–80]. Further studies have shown that this pathway is regulated by intracellular calcium levels and cAMP, and revealed additional drugs that may induce autophagy, including comparatively safe drugs that act on the brain, such as verapamil (an L-type calcium channel antagonist) and clonidine (an imidazoline receptor agonist that reduces cAMP levels) [81]. Proof-of-principle for these drugs has been provided in cell, *Drosophila* and zebrafish models of HD.

A recent study has shown tantalising effects of lithium in the treatment of amyotrophic lateral sclerosis (ALS) in humans and mouse models of this disease. Lithium carbonate (when given in addition to the benchmark drug riluzole), significantly delayed the onset of disability and death in human ALS patients, compared to those administered riluzole alone. Treatment of G93A ALS mice with lithium carbonate delayed death and onset of disease progression. Tissue collected from these mice showed an increase autophagic activity and a general decrease in disease phenotypes such as ubiquitin and alpha synuclein accumulation [82]. This significant correlation between autophagy induction and the delay of disease progression is compatible with our previous data showing that autophagy induction may protect against a range of neurodegenerative diseases caused by aggregate-prone proteins. Combination therapies utilising rapamycin plus a drug acting on an mTOR independent pathway may provide more effective treatments for neurodegeneration by providing an additive effect on autophagy induction while reducing the adverse side effects of rapamycin treatment alone.

## 9 Conclusion

Today 31 genes have been identified that play a specific role in the normal function of autophagy [83]. At least eight of these genes contribute to two conjugation reactions important for the formation of the autophagosome. As the understanding of autophagy has progressed, so have the available techniques and autophagy assays [84]. As our understanding of the autophagy machinery progresses, more advanced techniques will allow us to assess autophagy more precisely.

The progression of research in the field of autophagy has taken the understanding of autophagy from a means of survival during starvation to a possible means of treating neurodegeneration pharmacologically. Progress in the understanding of autophagy has emphasised the importance of autophagy in cellular homeostasis, adaptation to starvation, prevention of neurodegeneration in animal models, and the significance of its deficiency in the development of neurodegenerative disorders. The overall relevance of autophagy in disease far exceeds the breadth of this review. With its implicated roles ranging from viral infection and cancer to liver and cardiac diseases (reviewed in [73]), autophagy's importance in the maintenance of health is just beginning to be realised. Alongside understanding its implications in disease, future research will need to focus on a more complete characterisation of the autophagy machinery and how it responds to autophagy-regulating signalling pathways. This may provide further insights into the role that autophagy has in disease and how it may be possibly utilised as a therapeutic strategy.

## References

[1] Verhoef L.G., Lindsten K., Masucci M.G., Dantuma N.P. (2002). Aggregate formation inhibits proteasomal degradation of polyglutamine proteins. Hum. Mol. Genet..

[2] Takeshige K., Baba M., Tsuboi S., Noda T., Ohsumi Y. (1992). Autophagy in yeast demonstrated with proteinase-deficient mutants and conditions for its induction. J. Cell Biol..

[3] Egner R., Thumm M., Straub M., Simeon A., Schuller H.J., Wolf D.H. (1993). Tracing intracellular proteolytic pathways. Proteolysis of fatty acid synthase and other cytoplasmic proteins in the yeast *Saccharomyces cerevisiae*. J. Biol. Chem..

[4] Scott S.V., Baba M., Ohsumi Y., Klionsky D.J. (1997). Aminopeptidase I is targeted to the vacuole by a nonclassical vesicular mechanism. J. Cell Biol..

[5] Thumm M., Egner R., Koch B., Schlumpberger M., Straub M., Veenhuis M., Wolf D.H. (1994). Isolation of autophagocytosis mutants of *Saccharomyces cerevisiae*. FEBS. Lett..

[6] Tsukada M., Ohsumi Y. (1993). Isolation and characterization of autophagy-defective mutants of *Saccharomyces cerevisiae*. FEBS. Lett..

[7] Harding T.M., Morano K.A., Scott S.V., Klionsky D.J. (1995). Isolation and characterization of yeast mutants in the cytoplasm to vacuole protein targeting pathway. J. Cell Biol..

[8] Klionsky D.J., Cregg J.M., Dunn W.A., Emr S.D., Sakai Y., Sandoval I.V., Sibirny A., Subramani S., Thumm M., Veenhuis M., Ohsumi Y. (2003). A unified nomenclature for yeast autophagy-related genes. Dev. Cell.

[9] Ichimura Y., Kirisako T., Takao T., Satomi Y., Shimonishi Y., Ishihara N., Mizushima N., Tanida I., Kominami E., Ohsumi M., Noda T., Ohsumi Y. (2000). A ubiquitin-like system mediates protein lipidation. Nature.

[10] Mizushima N., Noda T., Yoshimori T., Tanaka Y., Ishii T., George M.D., Klionsky D.J., Ohsumi M., Ohsumi Y. (1998). A protein conjugation system essential for autophagy. Nature.

[11] Sou Y.S., Tanida I., Komatsu M., Ueno T., Kominami E. (2006). Phosphatidylserine in addition to phosphatidylethanolamine is an in vitro target of the mammalian Atg8 modifiers, LC3, GABARAP, and GATE-16. J. Biol. Chem..

[12] Ichimura Y., Imamura Y., Emoto K., Umeda M., Noda T., Ohsumi Y. (2004). In vivo and in vitro reconstitution of Atg8 conjugation essential for autophagy. J. Biol. Chem..

[13] Kuma A., Mizushima N., Ishihara N., Ohsumi Y. (2002). Formation of the approximately 350-kDa Apg12–Apg5.Apg16 multimeric complex, mediated by Apg16 oligomerization, is essential for autophagy in yeast. J. Biol. Chem..

[14] Mizushima N., Noda T., Ohsumi Y. (1999). Apg16p is required for the function of the Apg12p–Apg5p conjugate in the yeast autophagy pathway. EMBO. J..

[15] Suzuki K., Kirisako T., Kamada Y., Mizushima N., Noda T., Ohsumi Y. (2001). The pre-autophagosomal structure organized by concerted functions of APG genes is essential for autophagosome formation. EMBO. J..

[16] Kabeya Y., Mizushima N., Ueno T., Yamamoto A., Kirisako T., Noda T., Kominami E., Ohsumi Y., Yoshimori T. (2000). LC3, a mammalian homologue of yeast Apg8p, is localized in autophagosome membranes after processing. EMBO. J..

[17] Kuma A., Hatano M., Matsui M., Yamamoto A., Nakaya H., Yoshimori T., Ohsumi Y., Tokuhisa T., Mizushima N. (2004). The role of autophagy during the early neonatal starvation period. Nature.

[18] Komatsu M., Waguri S., Ueno T., Iwata J., Murata S., Tanida I., Ezaki J., Mizushima N., Ohsumi Y., Uchiyama Y., Kominami E., Tanaka K., Chiba T. (2005). Impairment of starvation-induced and constitutive autophagy in Atg7-deficient mice. J. Cell Biol..

[19] Komatsu M., Waguri S., Chiba T., Murata S., Iwata J., Tanida I., Ueno T., Koike M., Uchiyama Y., Kominami E., Tanaka K. (2006). Loss of autophagy in the central nervous system causes neurodegeneration in mice. Nature.

[20] Hara T., Nakamura K., Matsui M., Yamamoto A., Nakahara Y., Suzuki-Migishima R., Yokoyama M., Mishima K., Saito I., Okano H., Mizushima N. (2006). Suppression of basal autophagy in neural cells causes neurodegenerative disease in mice. Nature.

[21] Komatsu M., Waguri S., Koike M., Sou Y.S., Ueno T., Hara T., Mizushima N., Iwata J., Ezaki J., Murata S., Hamazaki J., Nishito Y., Iemura S., Natsume T., Yanagawa T., Uwayama J., Warabi E., Yoshida H., Ishii T., Kobayashi A., Yamamoto M., Yue Z., Uchiyama Y., Kominami E., Tanaka K. (2007). Homeostatic levels of p62 control cytoplasmic inclusion body formation in autophagy-deficient mice. Cell.

[22] Nezis I.P., Simonsen A., Sagona A.P., Finley K., Gaumer S., Contamine D., Rusten T.E., Stenmark H., Brech A. (2008). Ref(2)P, the *Drosophila melanogaster* homologue of mammalian p62, is required for the formation of protein aggregates in adult brain. J. Cell Biol..

[23] Juhasz G., Erdi B., Sass M., Neufeld T.P. (2007). Atg7-dependent autophagy promotes neuronal health, stress tolerance, and longevity but is dispensable for metamorphosis in *Drosophila*. Genes Dev..

[24] Simonsen A., Cumming R.C., Brech A., Isakson P., Schubert D.R., Finley K.D. (2008). Promoting basal levels of autophagy in the nervous system enhances longevity and oxidant resistance in adult *Drosophila*. Autophagy.

[25] Gordon P.B., Seglen P.O. (1988). Prelysosomal convergence of autophagic and endocytic pathways. Biochem. Biophys. Res. Commun..

[26] Goldstein L.S., Yang Z. (2000). Microtubule-based transport systems in neurons: the roles of kinesins and dyneins. Annu. Rev. Neurosci..

[27] Aplin A., Jasionowski T., Tuttle D.L., Lenk S.E., DunnJr W.A. (1992). Cytoskeletal elements are required for the formation and maturation of autophagic vacuoles. J. Cell Physiol..

[28] Webb J.L., Ravikumar B., Rubinsztein D.C. (2004). Microtubule disruption inhibits autophagosome-lysosome fusion: implications for studying the roles of aggresomes in polyglutamine diseases. Int. J. Biochem. Cell Biol..

[29] Ravikumar B., Acevedo-Arozena A., Imarisio S., Berger Z., Vacher C., O'Kane C.J., Brown S.D., Rubinsztein D.C. (2005). Dynein mutations impair autophagic clearance of aggregate-prone proteins. Nat. Genet..

[30] Kochl R., Hu X.W., Chan E.Y., Tooze S.A. (2006). Microtubules facilitate autophagosome formation and fusion of autophagosomes with endosomes. Traffic.

[31] Fass E., Shvets E., Degani I., Hirschberg K., Elazar Z. (2006). Microtubules support production of starvation-induced autophagosomes but not their targeting and fusion with lysosomes. J. Biol. Chem..

[32] Jahreiss L., Menzies F.M., Rubinsztein D.C. (2008). The itinerary of autophagosomes: from peripheral formation to kiss-and-run fusion with lysosomes. Traffic.

[33] Pandey U.B., Nie Z., Batlevi Y., McCray B.A., Ritson G.P., Nedelsky N.B., Schwartz S.L., DiProspero N.A., Knight M.A., Schuldiner O., Padmanabhan R., Hild M., Berry D.L., Garza D., Hubbert C.C., Yao T.P., Baehrecke E.H., Taylor J.P. (2007). HDAC6 rescues neurodegeneration and provides an essential link between autophagy and the UPS. Nature.

[34] Iwata A., Riley B.E., Johnston J.A., Kopito R.R. (2005). HDAC6 and microtubules are required for autophagic degradation of aggregated huntingtin. J. Biol. Chem..

[35] Hafezparast M., Klocke R., Ruhrberg C., Marquardt A., Ahmad-Annuar A., Bowen S., Lalli G., Witherden A.S., Hummerich H., Nicholson S., Morgan P.J., Oozageer R., Priestley J.V., Averill S., King V.R., Ball S., Peters J., Toda T., Yamamoto A., Hiraoka Y., Augustin M., Korthaus D., Wattler S., Wabnitz P., Dickneite C., Lampel S., Boehme F., Peraus G., Popp A., Rudelius M., Schlegel J., Fuchs H., Hrabe de Angelis M., Schiavo G., Shima D.T., Russ A.P., Stumm G., Martin J.E., Fisher E.M. (2003). Mutations in dynein link motor neuron degeneration to defects in retrograde transport. Science.

[36] King S.J., Schroer T.A. (2000). Dynactin increases the processivity of the cytoplasmic dynein motor. Nat. Cell Biol..

[37] LaMonte B.H., Wallace K.E., Holloway B.A., Shelly S.S., Ascano J., Tokito M., Van Winkle T., Howland D.S., Holzbaur E.L. (2002). Disruption of dynein/dynactin inhibits axonal transport in motor neurons causing late-onset progressive degeneration. Neuron.

[38] Puls I., Jonnakuty C., LaMonte B.H., Holzbaur E.L., Tokito M., Mann E., Floeter M.K., Bidus K., Drayna D., Oh S.J., BrownJr R.H., Ludlow C.L., Fischbeck K.H. (2003). Mutant dynactin in motor neuron disease. Nat. Genet..

[39] Laird F.M., Farah M.H., Ackerley S., Hoke A., Maragakis N., Rothstein J.D., Griffin J., Price D.L., Martin L.J., Wong P.C. (2008). Motor neuron disease occurring in a mutant dynactin mouse model is characterized by defects in vesicular trafficking. J. Neurosci..

[40] Hicke L. (2001). A new ticket for entry into budding vesicles-ubiquitin. Cell.

[41] Marchese A., Benovic J.L. (2001). Agonist-promoted ubiquitination of the G protein-coupled receptor CXCR4 mediates lysosomal sorting. J. Biol. Chem..

[42] Rocca A., Lamaze C., Subtil A., Dautry-Varsat A. (2001). Involvement of the ubiquitin/proteasome system in sorting of the interleukin 2 receptor beta chain to late endocytic compartments. Mol. Biol. Cell.

[43] Rotin D., Staub O., Haguenauer-Tsapis R. (2000). Ubiquitination and endocytosis of plasma membrane proteins: role of Nedd4/Rsp5p family of ubiquitin-protein ligases. J. Membr. Biol..

[44] Shenoy S.K., McDonald P.H., Kohout T.A., Lefkowitz R.J. (2001). Regulation of receptor fate by ubiquitination of activated beta 2-adrenergic receptor and beta-arrestin. Science.

[45] Williams R.L., Urbe S. (2007). The emerging shape of the ESCRT machinery. Nat. Rev. Mol. Cell Biol..

[46] Morita E., Sundquist W.I. (2004). Retrovirus budding. Annu. Rev. Cell Dev. Biol..

[47] Moberg K.H., Schelble S., Burdick S.K., Hariharan I.K. (2005). Mutations in erupted, the *Drosophila* ortholog of mammalian tumor susceptibility gene 101, elicit non-cell-autonomous overgrowth. Dev. Cell..

[48] Thompson B.J., Mathieu J., Sung H.H., Loeser E., Rorth P., Cohen S.M. (2005). Tumor suppressor properties of the ESCRT-II complex component Vps25 in *Drosophila*. Dev. Cell..

[49] Vaccari T., Bilder D. (2005). The *Drosophila* tumor suppressor vps25 prevents nonautonomous overproliferation by regulating notch trafficking. Dev. Cell.

[50] Skibinski G., Parkinson N.J., Brown J.M., Chakrabarti L., Lloyd S.L., Hummerich H., Nielsen J.E., Hodges J.R., Spillantini M.G., Thusgaard T., Brandner S., Brun A., Rossor M.N., Gade A., Johannsen P., Sorensen S.A., Gydesen S., Fisher E.M., Collinge J. (2005). Mutations in the endosomal ESCRTIII-complex subunit CHMP2B in frontotemporal dementia. Nat.Genet..

[51] Parkinson N., Ince P.G., Smith M.O., Highley R., Skibinski G., Andersen P.M., Morrison K.E., Pall H.S., Hardiman O., Collinge J., Shaw P.J., Fisher E.M. (2006). ALS phenotypes with mutations in CHMP2B (charged multivesicular body protein 2B). Neurology.

[52] Momeni P., Schymick J., Jain S., Cookson M.R., Cairns N.J., Greggio E., Greenway M.J., Berger S., Pickering-Brown S., Chio A., Fung H.C., Holtzman D.M., Huey E.D., Wassermann E.M., Adamson J., Hutton M.L., Rogaeva E., St George-Hyslop P., Rothstein J.D., Hardiman O., Grafman J., Singleton A., Hardy J., Traynor B.J. (2006). Analysis of IFT74 as a candidate gene for chromosome 9p-linked ALS-FTD. BMC. Neurol..

[53] Parkinson N., Ince P.G., Smith M.O., Highley R., Skibinski G., Andersen P.M., Morrison K.E., Pall H.S., Hardiman O., Collinge J., Shaw P.J., Fisher E.M. (2006). ALS phenotypes with mutations in CHMP2B (charged multivesicular body protein 2B). Neurology.

[54] Lee J.A., Beigneux A., Ahmad S.T., Young S.G., Gao F.B. (2007). ESCRT-III dysfunction causes autophagosome accumulation and neurodegeneration. Curr. Biol..

[55] Filimonenko M., Stuffers S., Raiborg C., Yamamoto A., Malerod L., Fisher E.M., Isaacs A., Brech A., Stenmark H., Simonsen A. (2007). Functional multivesicular bodies are required for autophagic clearance of protein aggregates associated with neurodegenerative disease. J. Cell Biol..

[56] Nara A., Mizushima N., Yamamoto A., Kabeya Y., Ohsumi Y., Yoshimori T. (2002). SKD1 AAA ATPase-dependent endosomal transport is involved in autolysosome formation. Cell Struct. Funct..

[57] Shirahama K., Noda T., Ohsumi Y. (1997). Mutational analysis of Csc1/Vps4p: involvement of endosome in regulation of autophagy in yeast. Cell Struct. Funct..

[58] Rusten T.E., Vaccari T., Lindmo K., Rodahl L.M., Nezis I.P., Sem-Jacobsen C., Wendler F., Vincent J.P., Brech A., Bilder D., Stenmark H. (2007). ESCRTs and Fab1 regulate distinct steps of autophagy. Curr. Biol..

[59] Dunn W.A. (1990). Studies on the mechanisms of autophagy: maturation of the autophagic vacuole. J. Cell Biol..

[60] Settembre C., Fraldi A., Jahreiss L., Spampanato C., Venturi C., Medina D., de Pablo R., Tacchetti C., Rubinsztein D.C., Ballabio A. (2008). A block of autophagy in lysosomal storage disorders. Hum. Mol. Genet..

[61] Jeyakumar M., Dwek R.A., Butters T.D., Platt F.M. (2005). Storage solutions: treating lysosomal disorders of the brain. Nat. Rev. Neurosci..

[62] Journet A., Chapel A., Kieffer S., Roux F., Garin J. (2002). Proteomic analysis of human lysosomes: application to monocytic and breast cancer cells. Proteomics.

[63] Eskelinen E.L., Tanaka Y., Saftig P. (2003). At the acidic edge: emerging functions for lysosomal membrane proteins. Trends Cell Biol..

[64] Futerman A.H., van Meer G. (2004). The cell biology of lysosomal storage disorders. Nat. Rev. Mol. Cell Biol..

[65] Dierks T., Schmidt B., Borissenko L.V., Peng J., Preusser A., Mariappan M., von Figura K. (2003). Multiple sulfatase deficiency is caused by mutations in the gene encoding the human C(alpha)-formylglycine generating enzyme. Cell.

[66] Cosma M.P., Pepe S., Annunziata I., Newbold R.F., Grompe M., Parenti G., Ballabio A. (2003). The multiple sulfatase deficiency gene encodes an essential and limiting factor for the activity of sulfatases. Cell.

[67] Webb J.L., Ravikumar B., Atkins J., Skepper J.N., Rubinsztein D.C. (2003). Alpha-Synuclein is degraded by both autophagy and the proteasome. J. Biol. Chem..

[68] Qin Z.H., Wang Y., Kegel K.B., Kazantsev A., Apostol B.L., Thompson L.M., Yoder J., Aronin N., DiFiglia M. (2003). Autophagy regulates the processing of amino terminal huntingtin fragments. Hum. Mol. Genet.

[69] Shibata M., Lu T., Furuya T., Degterev A., Mizushima N., Yoshimori T., MacDonald M., Yankner B., Yuan J. (2006). Regulation of intracellular accumulation of mutant Huntingtin by Beclin 1. J. Biol. Chem..

[70] Ravikumar B., Vacher C., Berger Z., Davies J.E., Luo S., Oroz L.G., Scaravilli F., Easton D.F., Duden R., O'Kane C.J., Rubinsztein D.C. (2004). Inhibition of mTOR induces autophagy and reduces toxicity of polyglutamine expansions in fly and mouse models of Huntington disease. Nat. Genet..

[71] Ravikumar B., Duden R., Rubinsztein D.C. (2002). Aggregate-prone proteins with polyglutamine and polyalanine expansions are degraded by autophagy. Hum. Mol. Genet..

[72] Noda T., Ohsumi Y. (1998). Tor, a phosphatidylinositol kinase homologue, controls autophagy in yeast. J. Biol. Chem..

[73] Rubinsztein D.C. (2006). The roles of intracellular protein-degradation pathways in neurodegeneration. Nature.

[74] Berger Z., Davies J.E., Luo S., Pasco M.Y., Majoul I., O'Kane C.J., Rubinsztein D.C. (2006). Deleterious and protective properties of an aggregate-prone protein with a polyalanine expansion. Hum. Mol. Genet..

[75] Taylor J.P., Tanaka F., Robitschek J., Sandoval C.M., Taye A., Markovic-Plese S., Fischbeck K.H. (2003). Aggresomes protect cells by enhancing the degradation of toxic polyglutamine-containing protein. Hum. Mol. Genet..

[76] Davies J.E., Sarkar S., Rubinsztein D.C. (2006). Trehalose reduces aggregate formation and delays pathology in a transgenic mouse model of oculopharyngeal muscular dystrophy. Hum. Mol. Genet..

[77] Sarkar S., Davies J.E., Huang Z., Tunnacliffe A., Rubinsztein D.C. (2007). Trehalose, a novel mTOR-independent autophagy enhancer, accelerates the clearance of mutant huntingtin and alpha-synuclein. J. Biol. Chem..

[78] Sarkar S., Floto R.A., Berger Z., Imarisio S., Cordenier A., Pasco M., Cook L.J., Rubinsztein D.C. (2005). Lithium induces autophagy by inhibiting inositol monophosphatase. J. Cell Biol..

[79] Berger Z., Ttofi E.K., Michel C.H., Pasco M.Y., Tenant S., Rubinsztein D.C., O'Kane C.J. (2005). Lithium rescues toxicity of aggregate-prone proteins in *Drosophila* by perturbing Wnt pathway. Hum. Mol. Genet..

[80] Sarkar S., Krishna G., Imarisio S., Saiki S., O'Kane C.J., Rubinsztein D.C. (2008). A rational mechanism for combination treatment of Huntington's disease using lithium and rapamycin. Hum. Mol. Genet..

[81] Williams A., Sarkar S., Cuddon P., Ttofi E.K., Saiki S., Siddiqi F.H., Jahreiss L., Fleming A., Pask D., Goldsmith P., O'Kane C.J., Floto R.A., Rubinsztein D.C. (2008). Novel targets for Huntington's disease in an mTOR-independent autophagy pathway. Nat. Chem. Biol..

[82] Fornai F., Longone P., Cafaro L., Kastsiuchenka O., Ferrucci M., Manca M.L., Lazzeri G., Spalloni A., Bellio N., Lenzi P., Modugno N., Siciliano G., Isidoro C., Murri L., Ruggieri S., Paparelli A. (2008). Lithium delays progression of amyotrophic lateral sclerosis. Proc. Natl. Acad. Sci. U. S. A..

[83] Kabeya Y., Kawamata T., Suzuki K., Ohsumi Y. (2007). Cis1/Atg31 is required for autophagosome formation in *Saccharomyces cerevisiae*. Biochem. Biophys. Res. Commun..

[84] Klionsky D.J., Abeliovich H., Agostinis P., Agrawal D.K., Aliev G., Askew D.S., Baba M., Baehrecke E.H., Bahr B.A., Ballabio A., Bamber B.A., Bassham D.C., Bergamini E., Bi X., Biard-Piechaczyk M., Blum J.S., Bredesen D.E., Brodsky J.L., Brumell J.H., Brunk U.T., Bursch W., Camougrand N., Cebollero E., Cecconi F., Chen Y., Chin L.S., Choi A., Chu C.T., Chung J., Clarke P.G., Clark R.S., Clarke S.G., Clave C., Cleveland J.L., Codogno P., Colombo M.I., Coto-Montes A., Cregg J.M., Cuervo A.M., Debnath J., Demarchi F., Dennis P.B., Dennis P.A., Deretic V., Devenish R.J., Di Sano F., Dice J.F., Difiglia M., Dinesh-Kumar S., Distelhorst C.W., Djavaheri-Mergny M., Dorsey F.C., Droge W., Dron M., Dunn W.A., Duszenko M., Eissa N.T., Elazar Z., Esclatine A., Eskelinen E.L., Fesus L., Finley K.D., Fuentes J.M., Fueyo J., Fujisaki K., Galliot B., Gao F.B., Gewirtz D.A., Gibson S.B., Gohla A., Goldberg A.L., Gonzalez R., Gonzalez-Estevez C., Gorski S., Gottlieb R.A., Haussinger D., He Y.W., Heidenreich K., Hill J.A., Hoyer-Hansen M., Hu X., Huang W.P., Iwasaki A., Jaattela M., Jackson W.T., Jiang X., Jin S., Johansen T., Jung J.U., Kadowaki M., Kang C., Kelekar A., Kessel D.H., Kiel J.A., Kim H.P., Kimchi A., Kinsella T.J., Kiselyov K., Kitamoto K., Knecht E., Komatsu M., Kominami E., Kondo S., Kovacs A.L., Kroemer G., Kuan C.Y., Kumar R., Kundu M., Landry J., Laporte M., Le W., Lei H.Y., Lenardo M.J., Levine B., Lieberman A., Lim K.L., Lin F.C., Liou W., Liu L.F., Lopez-Berestein G., Lopez-Otin C., Lu B., Macleod K.F., Malorni W., Martinet W., Matsuoka K., Mautner J., Meijer A.J., Melendez A., Michels P., Miotto G., Mistiaen W.P., Mizushima N., Mograbi B., Monastyrska I., Moore M.N., Moreira P.I., Moriyasu Y., Motyl T., Munz C., Murphy L.O., Naqvi N.I., Neufeld T.P., Nishino I., Nixon R.A., Noda T., Nurnberg B., Ogawa M., Oleinick N.L., Olsen L.J., Ozpolat B., Paglin S., Palmer G.E., Papassideri I., Parkes M., Perlmutter D.H., Perry G., Piacentini M., Pinkas-Kramarski R., Prescott M., Proikas-Cezanne T., Raben N., Rami A., Reggiori F., Rohrer B., Rubinsztein D.C., Ryan K.M., Sadoshima J., Sakagami H., Sakai Y., Sandri M., Sasakawa C., Sass M., Schneider C., Seglen P.O., Seleverstov O., Settleman J., Shacka J.J., Shapiro I.M., Sibirny A., Silva-Zacarin E.C., Simon H.U., Simone C., Simonsen A., Smith M.A., Spanel-Borowski K., Srinivas V., Steeves M., Stenmark H., Stromhaug P.E., Subauste C.S., Sugimoto S., Sulzer D., Suzuki T., Swanson M.S., Tabas I., Takeshita F., Talbot N.J., Talloczy Z., Tanaka K., Tanida I., Taylor G.S., Taylor J.P., Terman A., Tettamanti G., Thompson C.B., Thumm M., Tolkovsky A.M., Tooze S.A., Truant R., Tumanovska L.V., Uchiyama Y., Ueno T., Uzcategui N.L., van der Klei I., Vaquero E.C., Vellai T., Vogel M.W., Wang H.G., Webster P., Wiley J.W., Xi Z., Xiao G., Yahalom J., Yang J.M., Yap G., Yin X.M., Yoshimori T., Yu L., Yue Z., Yuzaki M., Zabirnyk O., Zheng X., Zhu X., Deter R.L. (2008). Guidelines for the use and interpretation of assays for monitoring autophagy in higher eukaryotes. Autophagy.

